# Use of the supraclavicular artery island flap for reconstruction of maxillofacial defects: a case report and literature review

**DOI:** 10.1186/s12893-020-00987-2

**Published:** 2021-04-14

**Authors:** J. F. Sheng, P. Tang, L. Y. Ma, Y. C. Cai, J. Hu, T. Xu, C. L. Liao, C. Deng, C. Li

**Affiliations:** 1 The Third Hospital of Mianyang & Sichuan Mental Health Center, Head, Neck and Maxillofacial Surgery & Thyroid Department, Mianyang, 621000 Sichuan China; 2 Sichuan Cancer Hospital & Institute, Head and Neck Surgery, No.55, Section 4, South Renmin Road, Chengdu, Sichuan 610041 People’s Republic of China

**Keywords:** Supraclavicular artery island flap, Maxillofacial, Flap, Case report

## Abstract

**Background:**

Free flaps are widely used in maxillofacial reconstruction; however, this approach was not feasible in the current case. It was not possible because the free flap method requires microvascular anastomosis expertise, which is difficult, time-consuming and costly.

**Case presentation:**

An 86-year-old woman suffered squamous cell carcinoma on the right side of her face, which resulted in a large soft-tissue defect. Here, we present a case of facial reconstruction from the inferior margin of the jaw to the top of the head. The size of the defect was 18.5 cm × 7.5 cm, which is rare for a patient of this age in the maxillofacial area. We used the supraclavicular artery island flap (SCAIFP) which measured 19.3 cm × 8.3 cm to repair the defect. After the operation, the flap survived without complications. Then, the patient was followed for 10 months and was satisfied with the aesthetic and functional results at the donor and recipient sites following the tumour resection. The tumour did not recur, and facial nerve function was preserved.

**Conclusion:**

Our results provide a new choice for the reconstruction of large defects of the head and face, and expand the potential applications of the SCAIFP.

## Background

Given the rise in the incidence of maxillofacial cutaneous malignant neoplasia, the provision of satisfactory treatment to repair the resulting facial defects can pose a significant surgical challenge. The free flap is an important component method of maxillofacial reconstruction that is widely used in China and is considered the gold standard of treatment. Our surgical team has previously used the free flap method to reconstruct large defects of the parotid and zygomatic regions [1]. Additionally, a double-free flap approach has been used to reconstruct giant soft tissue defects of the whole scalp [2, 3]. However, the free flap method requires microvascular anastomosis expertise, which is difficult, a long operating time, and maximum postoperative monitoring. As a result, it can be a troublesome task in patients with underlying conditions or who are not in good physical condition.

Recent advances in plastic surgery have enabled greater implementation of the application of the supraclavicular artery island flap (SCAIFP). Kozin and Emerick reported an island design utilizing a pedicled skin island into the neck with secondary de-epithelialization [4]. This flap is an easy- and quick-to-harvest flap that is colour matched, pliable and manageable. The SCAIFP has become very popular during the last few years [5]; however, only one case of large maxillofacial defect reconstruction using SCAIFP has been reported [6]. Recently, our surgical team successfully used the SCAIFP to repair a rare case of a large skin and soft tissue defect from the lower edge of the mandible to the top of the head where the defect size was 18.5 cm × 7.5 cm.

## Case presentation

**Fig. 1:**
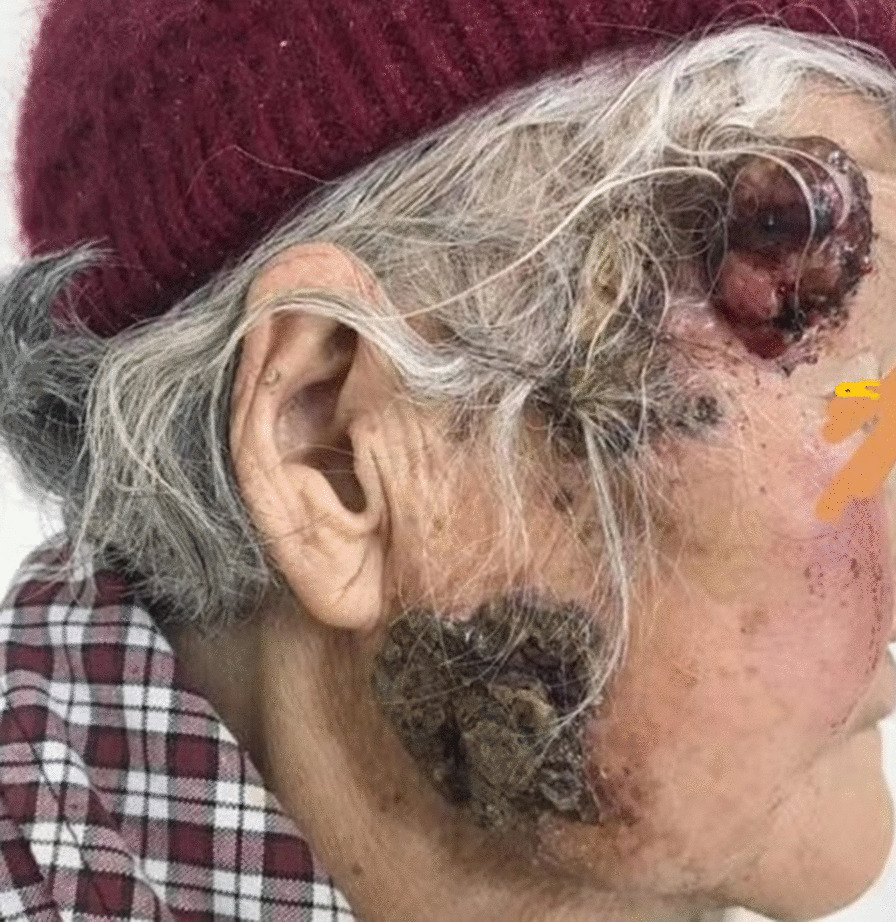
Preoperative view. An 86-year-old lady with neoplasm on her right face

The patient was an 86-year-old woman who suffered neoplasia on the right cheek and right frontotemporal area for 2 years. The patient had a history of lacunar infarction, cerebral insufficiency, hypertension, and diabetes. The facial neoplasm grew slowly for 2 years and accelerated in the last month before she was seen, with ulceration and crusting. The size of the neoplasia was 4.0 cm × 4.0 cm, 3.0 cm × 3.0 cm and 2.5 cm × 3.0 cm (Fig. 1).

Enhanced CT and MRI of the maxillofacial region were performed preoperatively. The surgery area was simulated using 3D image technology for the virtual surgery design and operation to be implemented. A safe resection range of 1.5 cm on the outer edge of the lesion was designed. One day before the surgery, the shape and range of the perforating branches were determined by Doppler ultrasound. The size and shape of the SCAIFP were designed to be elliptical and 15 cm × 7 cm in size.

**Fig. 2:**
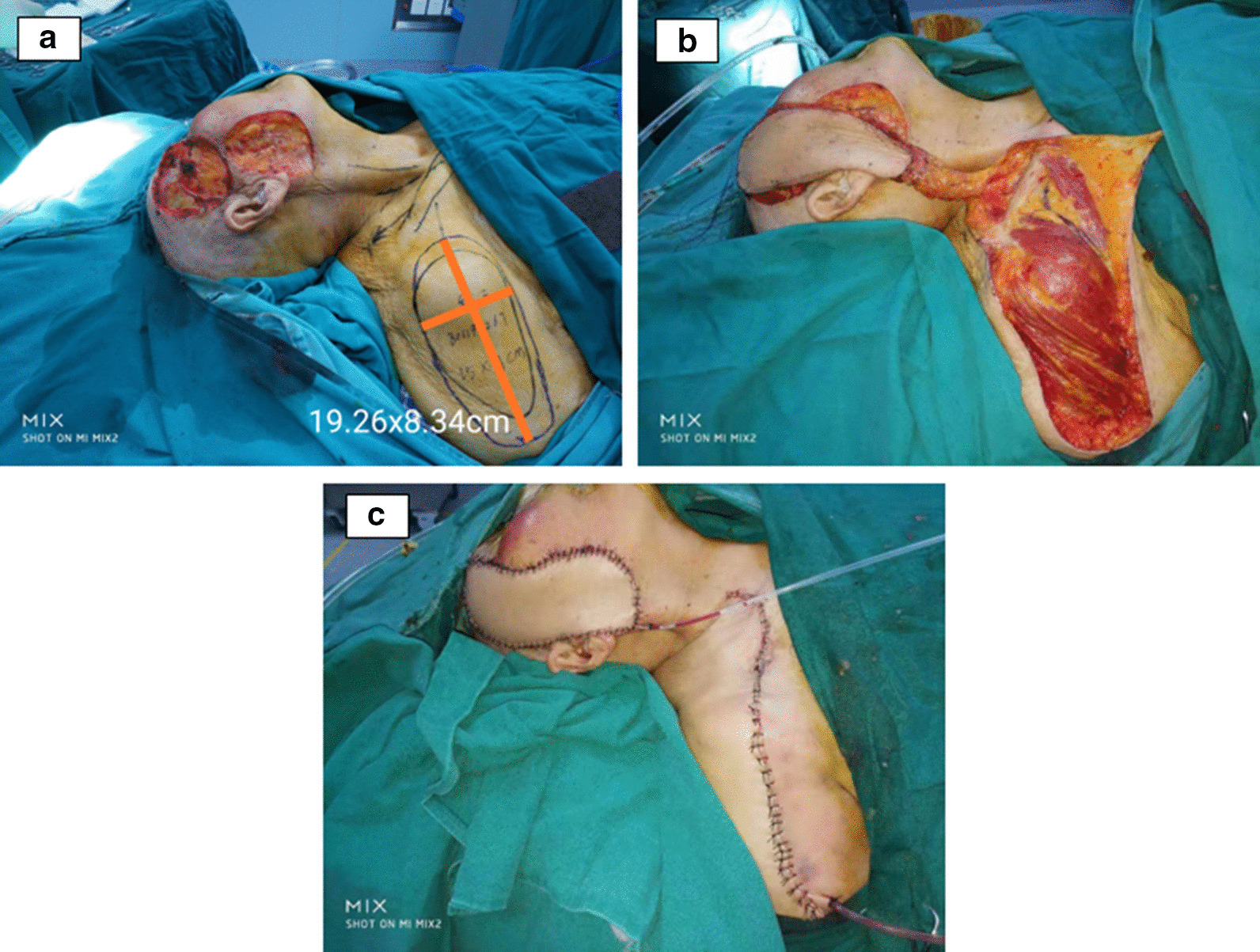
Intraoperative view. **a** The defect after the resection and the designed size of the flap was 19.26 cm × 8.24 cm. **b** The flap was fully free and released. **c** The recipient was and the donor was sutured

The maxillofacial tumour extended resection and supraclavicular flap repair operation was performed. The neoplasia was completely resected, and the facial nerve was preserved. The defect was approximately 18.5 cm × 7.5 cm. Pathological analysis of the frozen tissue showed low-medium differentiated squamous cell carcinoma with negative margins. Based on the position of the transverse carotid artery, the final flap size was 19.3 cm × 8.3 cm, which was larger than the initial flap design (15 cm × 7 cm). The larger size was a result of the defect being larger than initially expected and due to flap tension (Fig. 2). On the surface of the deltoid muscle, the flap was dissected from distal to proximal, and the superficial layer of the deep cervical fascia was reserved. The transverse carotid artery was cut off and ligated to enter the deep branches of the trapezius muscle to extend the reach. We used fluoroscopy to identify and protect the perforating branches. After the flap was harvested, a warm saline gauze was used for cover and protection, and the blood supply of the distal end of the flap was evaluated.

**Fig. 3:**
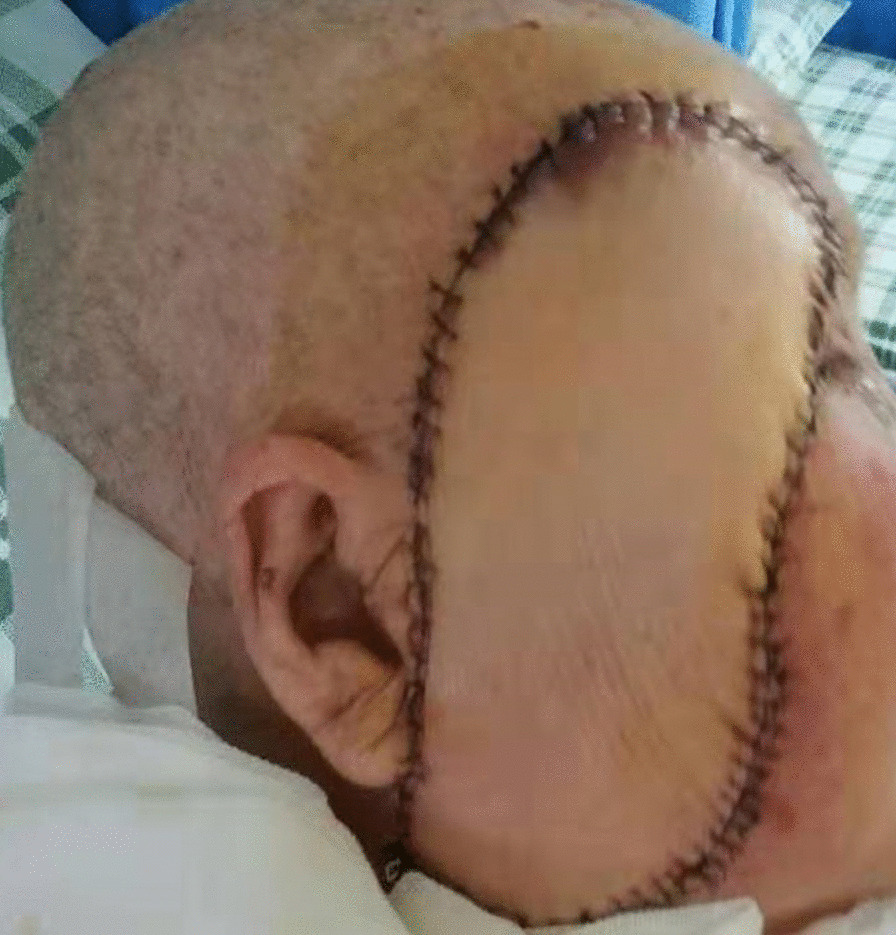
Postoperative view. The patient was discharged on postoperative day 7

**Fig. 4:**
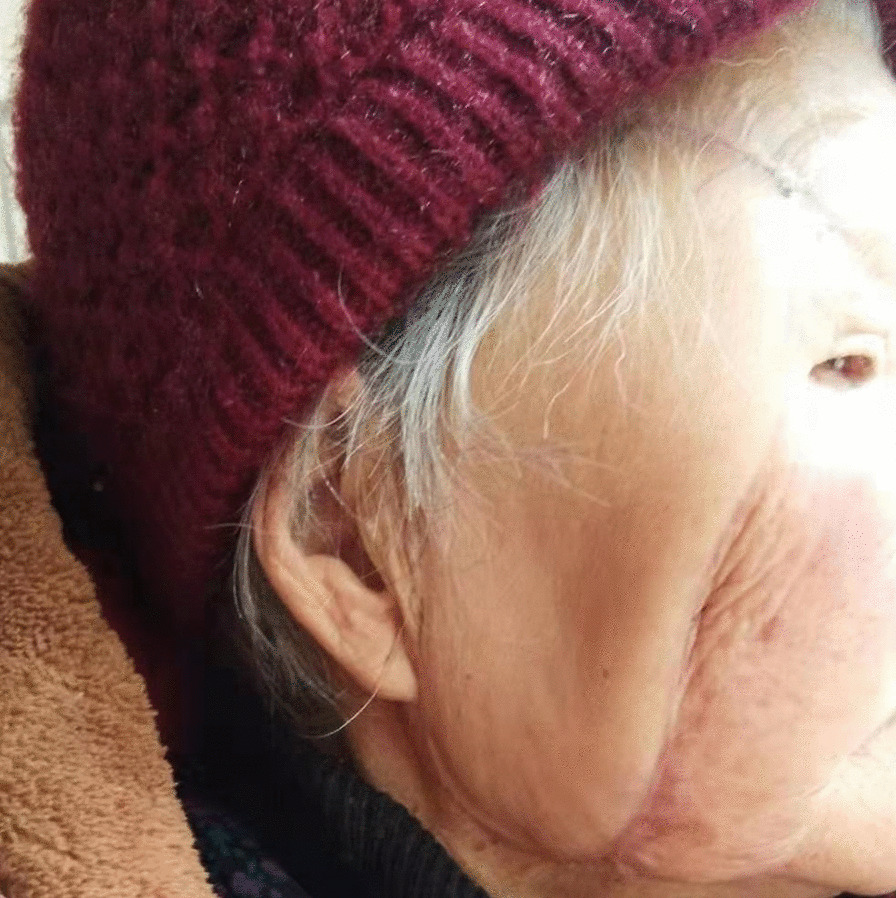
Postoperative view. The facial nerve function was well, the expression was natural on postoperative months 10

No complications were observed after the reconstruction. The flap survived entirely, and the donor area healed well. The patient was discharged on postoperative day 7 (Fig. 3), and at the 10-month follow-up coverage was stable with no tumour recurrence and the scar from the incision was natural. Upper limb abduction was 90° without dysfunction. Facial nerve function was well preserved, and facial expressions were natural and satisfactory for the patient (Fig. 4).

## Discussion

Reconstructing the original face of a patient is one of the most challenging aspects of plastic surgery. In addition to choosing from many different surgical techniques, the choice of the flap is also critically important for plastic surgeons during facial reconstruction. This particular case was unique because the maxillofacial local defect was large, occupying an area of 18.5 cm × 7.5 cm, and the defect reached the top of the forehead, making flap selection difficult. Additionally, the patient was elderly and had several underlying conditions. The duration and aggressiveness of the operation were reduced to maximize the potential for rapid recovery.

Over the past 20 years, the free flap method has been established as the optimal approach for reconstruction of the head and neck*.* The anterolateral thigh free flap is an ideal choice for large-scale soft tissue defects of the head and neck. In this case, the donor area could not be closed and sutured and would therefore require skin grafting.

The free flap is also a more expensive procedure [1], and since the patient came from a poor family, this was not a viable approach due to the high cost. As an alternative, the local pedicled flap can be used. The pectoralis major and latissimus dorsi, both local flaps, have a long history in the reconstruction of large head and neck defects. However, these muscles are thick and inflexible, which can present challenges to achieving satisfactory cosmetic results. Finally, we chose to use the SCAIFP after comprehensive consideration of the colour, thickness, size, rotation degree, vascular pedicle length, height, and condition of the donor area of the flap.

SCAIFP developments have increased its application in head and neck reconstruction [7, 8]. Granzow et al. [9] discussed the use of SCAIFP, which has largely been applied to thin and soft tissue defects and can be used as a first-line alternative to free flap reconstruction. The skin of the SCAIFP is very similar to the facial skin in colour, texture, thickness and characteristics of the hair. The size of the SCAIFP is between 4 to 12 cm wide and 20 to 30 cm long. Most authors suggest using Doppler guidance to locate the artery and collect flaps [10–12].

The skill of the surgeon allowed the flap preparation and primary focus resection to be completed without changes in body position. This prevented damage to the muscles and motor nerves of the shoulder [13] and had minimal impact on the function of the donor site [14, 15]. A series of reports from 349 cases of SCAIFP report the complete necrosis rate as 1.4% and the partial necrosis rate as 6.9% [16]. Moreover, the SCAIFP can be directly pulled and sutured, resulting in a hidden scar. Depending on the experience of the surgeon, it can take between 40 to 60 min to obtain the SCAIFP [4, 5]. In this case, the SCAIFP was obtained in 45 min. Postoperative monitoring is easier than with the free flap approach because it can be conveniently carried out in a basic hospital that is equipped for a range of clinical applications.

The SCAIFP approach used in this study had several associated limitations. First, it cannot be used in patients who require ligation of the transverse carotid artery. In patients with a previous history of neck surgery or neck radiotherapy, the transverse carotid artery and external jugular vein system should be carefully evaluated. Second, in patients with damage to the vascular system, the flap remains insufficient due to the large size of the defect (> 30 cm length).

## Conclusions

The SCAIFP is a thin skin flap with an appropriate texture that makes it applicable to maxillofacial reconstruction. Based on the available reports in Medline, we found that the largest repair area of SCAIFP has been 10 cm × 20 cm [5], and the highest repair height has been in the temporal area [17]. However, SCAIFP has not yet been reported to repair large defects of the head and face that reach the top of the forehead. Our results provide a new choice for the reconstruction of large defects of the head and face and expand the potential applications of the SCAIFP. We believe that the free flap plays a key role in the reconstruction of large defects of the head and neck; however, more research is needed to investigate the approach.

## Data Availability

Data sharing does not apply to this article, as no datasets were generated or analysed during the current study.

## Abbreviation

SCAIFP: Supraclavicular artery island flap
